# Injury Characteristics and Associated Factors in Federated Artistic Skaters: A Cross-Sectional Survey

**DOI:** 10.3390/healthcare14070951

**Published:** 2026-04-05

**Authors:** Nerea Blanco-Martínez, Daniel González-Devesa, Isabel Domingo Díaz-Malaguilla, Carlos Ayán-Pérez

**Affiliations:** 1 Departamento de Didácticas Especiáis, Universidade de Vigo, 36005 Pontevedra, Spain; n.blanco@uvigo.gal (N.B.-M.); cayan@uvigo.es (C.A.-P.); 2 Well-Move Research Group, Galicia Sur Health Research Institute (IIS Galicia Sur), SERGAS-UVIGO, 36310 Vigo, Spain; 3 Grupo de Investigación en Actividad Física, Educación, y Salud (GIAFES), Universidad Católica de Ávila, 05005 Ávila, Spain; 4 Facultad de Ciencias de la Educación y del Deporte, Universidad de Vigo, 36005 Pontevedra, Spain

**Keywords:** roller skating, athletes, sprains and strains, female, menarche

## Abstract

**Background**: Artistic skating involves high mechanical demands (e.g., jumps and spins) that may increase injury frequency, yet discipline-specific evidence remains limited. Objective: The aim of this study is to describe injury characteristics in federated artistic skaters and to explore factors associated with injury frequency. **Methods**: A descriptive cross-sectional study was conducted using an online survey distributed to federated clubs. Eligible participants practiced show and/or free skating, were federated athletes, and had competed at least once in an official competition. **Results**: Fifty artistic skaters participated (90% women; age 18.37 ± 3.58 years), recruited from 13 clubs; 28% competed in show, 30% in free, and 42% in both disciplines. All participants reported at least one injury; 58% occurred on the right side, and injuries most frequently affected the lower limb. The most commonly reported injury types were muscle injuries (26%), sprains (20%), tendon injuries (18%), and single-bone fractures (12%). Jumping was the most frequent action at the time of injury (40%), followed by spins (20%). Injuries most often occurred on parquet (42%) and polished concrete (38%), and 54% of athletes required physiotherapy. Time-loss was ≤7 days in 44% of cases, while 28% reported >28 days. A significant association was found between time since first menstruation and having sustained >1 injury (*p* = 0.034). No significant differences were observed in other demographic variables, training/competition characteristics, or preventive practices between groups (*p* > 0.05). **Conclusions**: Injuries in federated Spanish artistic skaters were predominantly lower-limb and commonly occurred during jumping, frequently requiring physiotherapy and, in a substantial proportion, leading to prolonged time-loss. Injury frequency was associated with time since first menstruation, while training load indicators and preventive practices did not differ between skaters with one versus multiple injuries.

## 1. Introduction

Participation in sports activities has been associated with substantial physical, psychological, and social benefits, including improvements in physical fitness and body composition, a lower prevalence of anxiety and depressive symptoms, and greater overall well-being across the lifespan [1]. However, sports participation is also accompanied by an increased risk of injury and constitutes a relevant public health concern during this developmental stage [2]. Injuries may increase the likelihood of sport dropout and reduce physical activity levels, thereby diminishing the health benefits derived from regular practice [3]. Moreover, sports-related injuries may lead to negative musculoskeletal consequences in the medium and long term, such as persistent pain, structural alterations, and a higher probability of developing degenerative conditions [4,5]. These consequences may, in turn, substantially interfere with quality of life [6]. To date, most epidemiological research on sports injuries has focused on widely practiced disciplines such as football [7], basketball [8], volleyball [9], and gymnastics [10].

Sports characterized by high technical and aesthetic demands have been described as disciplines with a specific injury profile, in which overuse injuries associated with repetitive movements predominate [11]. In this context, artistic roller skating involves substantial physical demands derived from the discipline’s technical and artistic requirements, making injuries a relevant concern for both athletes and coaches [12]. Moreover, among sports practiced on wheels, artistic roller skating has been identified as the discipline with the highest risk of accidents [13]. This sport is also typically performed in a highly structured and demanding training environment. Such conditions may increase psychological pressure on young athletes and further contribute to injury risk [14].

Effective implementation of injury prevention strategies in sport requires comprehensive epidemiological knowledge [15]. Such knowledge allows not only the estimation of injury incidence, but also the identification of sport-specific injury patterns, including anatomical location, injury type and severity, as well as underlying mechanisms and contextual risk factors [16]. This information helps identify modifiable factors involved in injury occurrence and supports the design of prevention strategies tailored to the specific demands of each sport [17]. Consequently, epidemiological studies of this kind remain necessary for several reasons, including informing potential participants about injury risk, providing valuable data to optimize healthcare provision in sport, and contributing to the development of safer participation environments [18]. Moreover, the availability of robust epidemiological evidence is essential for sports federations and international organizations to effectively guide policies and prevention programs aimed at protecting athletes’ health [19]. In addition, it is relevant to examine whether athletes sustain more than one injury during a competitive season, as repeated injury episodes may reflect persistent exposure to risk factors, incomplete recovery, or suboptimal return-to-sport decisions [20,21].

Despite the recognized importance of epidemiological research for the development of effective injury prevention strategies [15], studies specifically examining injury recurrence and its associated factors in federated artistic roller skaters remain scarce [22]. This lack of evidence limits the identification of consistent injury patterns and hampers the design of prevention programs adapted to the specific demands of this sport. Therefore, the aim of the present study was to describe the characteristics of sports-related injuries and to analyze the related factors in federated artistic roller skaters over the course of a competitive season.

## 2. Materials and Methods

### 2.1. Participants

Participants in this study were recruited through advertisements disseminated on social media and via email invitations sent to artistic roller skating federations and clubs in Spain. Eligibility criteria included: (a) practiced the show or free skating modality; (b) were federated athletes; and (c) had participated in at least one national and/or international competition during the last season. Previous injury history, irrespective of the number of injuries sustained, was not considered an inclusion or exclusion criterion.

Given the exploratory nature of the study and the limited accessibility of this specific federated population, no formal a priori calculation of sample size for hypothesis testing was conducted. Nevertheless, the final sample size was comparable to those reported in previous studies involving competitive skaters [23,24,25]. All participants were informed about the study procedures and provided electronic informed consent. The data analyzed in the present study were originally collected in the context of a Bachelor’s degree thesis at the Universidade de Vigo. In this context, the survey was conducted in accordance with the guidance and recommendations of the Ethics Committee of the Faculty of Education and Sports Sciences at the Universidade de Vigo (code: 04-090226), and the present study was conducted in accordance with the Declaration of Helsinki (2013).

### 2.2. Procedures

An online survey was sent to participants across several regions of Spain via social media or email link during the 2024–2025 season, asking them to report the injuries sustained throughout the previous season. A skating-specific questionnaire was developed, based on an instrument previously used in similar study [26], and adapted to the characteristics of this modality. The survey included questions on: (a) sociodemographic and anthropometric variables; (b) sport background and competitive profile; (c) training exposure; (d) context at the time of the injury; and (d) injury history and characteristics. A combination of question formats (multiple-choice, Likert-scale, and open-ended items) was used to address these themes. Injury was defined as any physical complaint sustained during sport participation that resulted in the athlete being unable to fully participate in future training or competition [27]. Injury frequency was considered when an athlete sustained more than one injury during training or competition within the same season.

To adapt the questionnaire to the characteristics of artistic roller skating, one researcher with prior experience in sports injury research and another researcher, a former skater, discussed which sport-specific actions should be included as potentially injury-related. They also considered relevant characteristics of competition facilities (e.g., floor type) and contextual factors specific to the different disciplines of the sport. The resulting questionnaire was pilot-tested with two active skaters to assess its clarity and suitability for the purpose of this research. All data were collected anonymously, with no personal identifiers, to ensure participant privacy.

### 2.3. Statistical Analysis

Statistical analyses were performed using IBM SPSS Statistics, version 27.0 (IBM Corp., Armonk, NY, USA). Continuous variables were reported as mean ± standard deviation (SD) when normally distributed or as median and interquartile range (IQR) when not normally distributed, and categorical variables as absolute and relative frequencies, n (%). The normality of the distribution of continuous variables was assessed using the Shapiro–Wilktest. Differences between two independent groups were assessed using the Mann–Whitney U test for non-normally distributed data and the independent samples *t*-test for normally distributed data. Categorical variables were analyzed using a Chi-Square Test of Independence and Cramer’s V test or Fisher’s exact test when the contingency table was 2 × 2. A Cramer’s V value that was less than 0.20 was observed as having a weak association, between 0.20 and 0.49 as a moderate association, and values above 0.49 as a strong association [28]. Odds ratios (ORs) with 95% confidence intervals (95% CIs) were also calculated to quantify the strength of associations. Statistical significance was set at *p* < 0.05.

## 3. Results

A total of 50 athletes (90% women) participated in the study (mean age = 18.37 ± 3.58 years; height = 163.20 ± 8.18 cm; body mass = 56.88 ± 8.26 kg). Participants were recruited from 13 federated clubs. The sample was divided into show discipline (28%), free skating (30%), and both disciplines (42%). Table 1 shows the participants’ characteristics by injury frequency. A significant association was observed between time since first menstruation and having experienced more than one injury (*p* = 0.034), with a moderate effect size (Cramer’s V= 0.416). No significant differences were found for the remaining demographic variables (*p* > 0.05).

**Figure 1:**
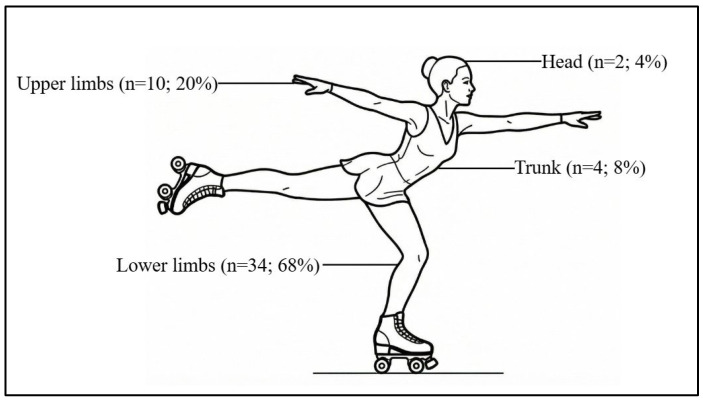
Anatomical location of injuries.

All participants included in this study had sustained at least one injury. Overall, 58% (n = 29) of injuries occurred on the right side of the body and 42% (n = 21) on the left side, with lower-limb injuries being the most frequent (Figure 1). Within the lower limbs, the knee was the most frequently affected anatomical site (52.9%), followed by the ankle (20.6%) and the thigh (17.6%), whereas in the upper limbs, injuries were mainly located at the wrist (50%) and the arm (40%). The most frequently reported injuries were muscle injuries (26%) and sprains (20%), followed by tendon injuries (18%) and single-bone fractures (12%). Less common injuries included dislocation/subluxation, multiple-bone fractures, laceration, ligament rupture, and cartilage separation. Participants were also asked how long they were unable to return to training following the injury. Overall, 18% (n = 9) reported being unable to train for 1–3 days, 26% (n = 13) for 4–7 days, and 28% (n = 14) for 8–28 days, and the remaining 28% (n = 14) reported being unable to train for more than 28 days.

The actions most frequently reported at the time of injury were jumping (40%), followed by spins (20%) and forward crossovers (14%). Regarding the surface on which injuries occurred, parquet was the most common (42%), followed by polished concrete (38%). In addition, 54% of participants reported requiring physiotherapy after the injury.

All injured athletes combined their sport practice with academic activities. In this regard, 26% (n = 13) of participants reported that they had to interrupt their academic schedule. However, 96% reported that the injury did not negatively affect their academic performance.

Table 2 and Table 3 summarize potential risk factors associated with injury frequency. No significant between-group differences were observed in training and competition characteristics or in preventive practices (*p* > 0.05).

## 4. Discussion

The aim of the present study was to analyze the characteristics and frequency of sports-related injuries in federated artistic roller skaters during a competitive season. The main findings indicate that all athletes experienced at least one injury throughout the season, with a clear predominance of lower-limb injuries, particularly muscle injuries and sprains, and a high proportion of injury episodes occurring during the execution of jumps. Overall, these results confirm that injury frequency represents a relevant issue in this discipline and provide valuable information for both sports and healthcare professionals, as well as for athletes themselves, to support the development of sport-specific injury prevention strategies.

Regarding injury typology, the high frequency of lower-limb involvement observed in our study is consistent with previous findings in artistic roller skating (53.1%) [22,29] and in other similar technical–artistic disciplines, such as figure skating (around 66%) [30,31]. Knee injuries emerged as the most prevalent among lower-limb injuries (52.9%), followed by those affecting the ankle (20.6%), while injuries to the wrist were the most common within the upper limbs, which is consistent with previous findings reported in Portuguese female artistic roller skaters by Cabo et al. [22]. In contrast, Dhodapkar et al. [32], in an epidemiological analysis of injuries across skating sports, reported different anatomical distributions, with injuries occurring predominantly in the head/face/neck in ice skating (34.5%) and in the shoulder/arm/elbow/wrist in inline and roller skating (53.0% and 49.9% respectively). Nevertheless, the high proportion of muscle injuries, sprains and tendon injuries observed in our study may be explained by the combination of technically demanding explosive actions performed repeatedly within extreme ranges of motion, together with high demands on neuromuscular control and functional capacity [33]. Furthermore, the fact that jumps were the most frequently involved action in injury mechanisms supports the notion that these technical gestures represent one of the main risk factors, particularly during the landing phase [12,34] and when performed repeatedly on relatively rigid surfaces [35], such as parquet or concrete, which were predominant in the present study.

The injury severity observed in the present study suggests a moderate to high level of impact, as more than half of the athletes were unable to train for over one week and nearly one third for more than four weeks. These findings are consistent with previous reports in artistic skaters describing recovery periods ranging from several weeks to several months [31], as well as with evidence from other roller sports, such as rink hockey [36,37], thereby highlighting the substantial sporting consequences of injuries in this discipline. Moreover, the interruption of academic activities reported by a relevant proportion of athletes indicates that, during this developmental stage, the impact of injuries may extend beyond sports participation, with potential implications for daily functioning, psychosocial well-being, and future health trajectories [38,39]. In this regard, particular attention should be paid to injuries that may recur, as repeated episodes could contribute to chronicity or even disability, with potential consequences for functionality and quality of life [40,41].

An interesting finding of the present study is that time since menarche was the only variable significantly associated with injury frequency. Although this result may be in line with previous evidence suggesting that the process of biological maturation plays an important role in injury vulnerability among female athletes [42], It should be interpreted with caution given the small sample size, the multiple testing performed, and the retrospective, self-reported nature of the questionnaire used in this study. In this context, hormonal changes during puberty, together with modifications in body composition and alterations in neuromuscular and ligamentous properties, may influence the ability to adapt to training loads and tissue recovery processes [43,44,45]. Furthermore, early menarche has been described as a moderate risk factor for the development of musculoskeletal injuries [44], and subsequently, female athletes in postmenarcheal stages exhibit greater susceptibility to injury compared with those in premenarcheal phases [43].

With respect to training-related variables, no significant differences were observed between athletes with single and multiple injuries in terms of training frequency, weekly training volume, session duration, competition exposure, or choreography length, suggesting that injury frequency in artistic roller skating may not be adequately explained by basic quantitative measures of training exposure alone. In this regard, our findings are consistent with those reported by Rebelo et al. [12], who showed that injury risk in female artistic roller skaters is more closely related to training load structure and content—particularly jump volume, mechanical external load, and training monotony— than to total training hours, although evidence from other sports suggests that total training volume may also play a relevant role in injury risk [46,47].

Finally, regarding the implementation of preventive strategies, no significant associations were observed in the present study between injury frequency and either the performance or the characteristics of warm-up, stretching, or strength training routines. These findings suggest that the mere inclusion of such practices may not be sufficient to reduce injury risk, which is consistent with the multifactorial nature of sports injuries [48]. However, the variables assessed in the present study captured only the presence and general characteristics of these preventive practices and did not provide information regarding their quality, specificity, progression, or level of supervision. Moreover, it was not possible to determine whether these activities were performed with the explicit objective of reducing injury risk or were simply part of routine or standardized training practices. Consequently, no causal relationship between these practices and a reduction in injury risk can be inferred from the present findings. Accordingly, the potential effectiveness of these practices may depend less on their mere presence and more on how they are structured, supervised, and tailored [49], which reinforces the need to interpret injury occurrence in artistic roller skating from a multifactorial perspective that also takes into account previous injuries, training load, competitive demands, and the use of sports equipment, as these factors have been associated with a higher frequency of injury episodes in this discipline [29].

From a practical perspective, these findings suggest that injury prevention in artistic roller skating should focus not only on training volume, but also on jump-related demands, training load structure, and the supervised implementation of sport-specific preventive routines targeting lower-limb control and landing mechanics. A key strength of the present study is that it contributes to a still limited body of evidence by characterizing the injury pattern in artistic roller skating. Nevertheless, although the data obtained provide information of interest for the discipline, the present study is not without limitations that should be considered when interpreting the findings. First, objective data on training and competition exposure time were not recorded, which prevents the calculation of injury frequency and limits a more precise analysis of associated risk factors. Second, a self-reported questionnaire was used, meaning that the collected information relied on participants’ recall and may therefore be subject to memory bias. Although the questionnaire was based on a previously used instrument, its psychometric properties were not assessed in this study, and the voluntary nature of participation may also have introduced selection bias, as athletes with health-related problems or a higher injury burden may have been more likely to respond. Third, injuries were not verified through medical reports or clinical records, which could affect the accuracy of the classification and characterization of injury episodes. Finally, the sample consisted exclusively of federated Spanish skaters from a specific region and competitive level, which limits the generalizability and international applicability of the findings to other populations, sporting contexts, and countries.

## 5. Conclusions

The practice of artistic roller skating may be considered potentially injurious, as all athletes included in the present study experienced at least one injury during the competitive season, regardless of the discipline practiced, highlighting the high injury burden associated with this sport. The most frequent injuries were muscle injuries and sprains, mainly affecting the lower limbs and largely associated with jumping actions, with the knee being the most commonly injured site, followed by the ankle, and the wrist in the upper limb. In this context, these results may inform more tailored preventive approaches, particularly those addressing the demands of jumping, the management of training exposure, and potential vulnerability during menarche. Future studies with larger sample sizes are needed to jointly examine these variables together with training load structure and content, in order to confirm the factors associated with injury risk in this discipline and to contribute to the development of specific prevention strategies.

## Figures and Tables

**Table 1:** Demographic characteristics of the participants.

	1 Injury (n = 21)	>1 Injury (n = 29)	*p-Value*	*Cramer’s V*
Sex (n; %)			0.638	0.122
*Male*	3 (14.29)	2 (6.9)		
*Female*	18 (85.71)	27 (93.10)		
Age (years)	17.78 ± 3.50	18.81 ± 3.64	0.336	
Years of practice	10.88 ± 3.11	12.34 ± 2.99	0.102	
Height (cm)	162 [8]	164 [5]	0.643	
Weight (kg)	56 [13]	60 [11]	0.296	
Body mass index (kg/m^2^)	20.50 ± 2.71	21.89 ± 2.37	0.067	
Time since first menstruation			0.034	0.416
*Never*	5 (23.81)	2 (6.90)		
*<1 year*	3 (14.29)	0		
*1–2 years*	2 (9.52)	2 (6.90)		
*>2 years*	11 (52.38)	25 (86.21)		
Discipline (n; %)			0.983	0.027
*Free skating*	6 (28.57)	9 (31.03)		
*Show*	6 (28.57)	8 (27.59)		
*Both*	9 (42.86)	12 (41.38)		

Note: Continuous variables are reported as mean ± SD for normally distributed data and median [IQR] for non-normally distributed data. Categorical variables are reported as n (%). Between-group differences were assessed using the independent samples *t*-test or Mann–Whitney U test for continuous variables, and the Pearson chi-square test or Fisher’s exact test, as appropriate, for categorical variables. Effect sizes for categorical comparisons are expressed as Cramér’s V.

**Table 2:** Training and competition characteristics by injury frequency.

	1 Injury (n = 21)	>1 Injury (n = 29)	*p-Value*	*Cramer’s V*
Season period			0.275	0.227
*Preseason*	5 (23.81)	13 (44.83)		
*Competitive season*	13 (61.90)	14 (48.28)		
*Postseason*	3 (14.29)	2 (6.89)		
Training days/week			0.864	0.257
*1*	1 (4.77)	1 (3.45)		
*2*	3 (14.29)	2 (6.90)		
*3*	3 (14.29)	4 (13.79)		
*4*	10 (47.62)	12 (41.38)		
*5*	3 (14.29)	7 (24.14)		
*≥6*	1 (4.77)	3 (10.34)		
Training hours/week	7.64 ± 3.52	7.67 ± 3.94	0.978	
Training session duration (minutes)	120 [83]	90 [30]	0.185	
Number competitions before injury (n)	1.5 [2.75]	1 [3]	0.413	
Choreography duration (minutes)	4 [2.72]	3.5 [2.5]	0.524	

Note: Continuous variables are reported as mean ± SD for normally distributed data and median [IQR] for non-normally distributed data. Categorical variables are reported as n (%). Between-group differences were assessed using the independent samples *t*-test or Mann–Whitney U test for continuous variables, and the Pearson chi-square test or Fisher’s exact test, as appropriate, for categorical variables. Effect sizes for categorical comparisons are expressed as Cramér’s V.

**Table 3:** Injury context and prevention-related practices according to injury frequency.

	1 Injury (n = 21)	>1 Injury (n = 29)	*p-Value*	*OR (95% CI)*	*Cramer’s V*
Warm-up in all sessions (yes)	16 (76.19)	22 (75.86)	0.999	0.98 (0.26–3.66)	0.004
Warm-up duration			0.117		0.343
*5–10 min*	8	15			
*10–15 min*	4	7			
*>15 min*	8	3			
Stretching in all sessions (yes)	16 (76.19)	22 (75.86)	0.999	0.98 (0.26–3.66)	0.004
Stretching (duration)			0.642		0.183
*5–10 min*	12	19			
*10–15 min*	5	3			
*>15 min*	1	2			
Strength & conditioning coach (yes)	6 (28.57)	6 (20.69)	0.738	0.65 (0.18–2.41)	0.091
Doing strength and conditioning training before injury (yes)	12 (57.14)	14 (48.28)	0.578	0.70 (0.23–2.17)	0.088
Injury situation			0.686	0.69 (0.13–3.83)	0.060
*Official competition*	3 (14.29)	3 (10.34)			
*Training*	18 (85.71)	26 (89.66)			

Note: Categorical variables are expressed as absolute and relative frequencies, n (%). Between-group differences were analyzed using Pearson’s chi-square test or Fisher’s exact test, as appropriate. Odds ratios (ORs) with 95% confidence intervals (CIs) are reported for binary variables. Cramer’s V is provided as a measure of effect size for categorical comparisons.

## Data Availability

The data supporting the findings of this study are available from the corresponding author upon reasonable request.
